# Toxic Properties of Sassafras

**Published:** 1885-12

**Authors:** John Bartlett

**Affiliations:** 482 North Clark Street; Chicago


THE CHICAGO

### Medical Journal and Examiner.

Vol. LI. DECEMBER, 1885. No. 6.

### Original Communications.

### Remarks on the Toxic Properties of Sassafras. *By* John Bartlett, m. d., Chicago.

[Read before the Chicago Gynecological Society, Noveember 27th, 1885.]

I wish this evening to call attention to a possible property of a medicine regarded up to a very recent date as almost inert.

Sassafras was discovered in Florida by the Spaniards and named by the French in 1562. It was used by them in association with other native herbs as a remedy for malarial diseases. For years past, though occasionally prescribed in combination in rheumatism and syphilis, and regarded as possessing diuretic, diaphoretic and tonic properties, it had become to be looked up as but little efficient. So that by referring to such books as were within my reach, namely, Motherby, 1785, Parr, 1809, Eberle, Trousseau, Mitchell, Warring, Stillé, Ringer, Bartholow, Phillips, Wood, Fluckeger, Farquharson, Brunton, Wormly and Blyth, and the U. S. Dispensatory, National Dispensatory, Christison's and King's Dispensatory, I can find no mention of the possession by sassafras of any decided therapeutical or noxious power.

More than twenty years ago Dr. Thompson, of Tennessee, stated that sassafras was an antidote to henbane and tobacco; and later, in 1870, Dr. Lyle, of Indiana, declared that he had used the oil of sassafras in a case of stramonium poisoning with the happiest results. Dr. Lyle affirmed that sassafras had power to destroy all insect life, and was an effectual antidote to the venom of the copperhead snake. In 1883 we find that Dr. Hinton claimed that sassafras tea was almost a specific for the rash produced by poison oak.

Recently paragraphs have appeared in the medical journals, in which it is stated that sassafras is not the innocent agent that it has been supposed to be, but that in reality it has violent toxic properties. This statement is made upon the authority of Dr. Charles L. Hill, from whose paper read before the 86th session of the Medical and Chirurgical Faculty of the State of Maryland, in April, 1884, the following report is extracted:

"A case of poisoning by the oil of sassafras, that once came within my knowledge, proved that it possesses far more active properties than is generally supposed, and I have been able to demonstrate by experiment on the lower animals that, instead of being a harmless, inert drug, it is a strong nervous sedative, anodyne and soporific, and in over-doses, a dangerous narcotic poison. A policeman, attracted by the sound of a falling window and other suspicious noises proceeding from a gentleman's office, entered the room to ascertain the cause. He found no one present but a boy, who was lying unconscious on the floor. He took him at once to the station-house, where I saw him shortly afterward. The officers had already diagnosed his case as one of opium-poisoning, and were vigorously striving to keep him awake by walking, flogging and such other means as are usually resorted to in these emergencies. His stupor was profound and he no longer made an attempt to walk, but was literally dragged about in their efforts to revive him. He spoke occasionally, but only to beg them to allow him to sleep. He was in a condition of great relaxation; skin covered with a profuse perspiration; countenance pallid; pulse rapid, but weak and thready. His pupils were *normal*, and there was a strong odor of sassafras in his breath. As quickly as possible an emetic was administered, which produced a copious emesis, redolent with the odor of sassafras, with drops of the undissolved oil floating in the liquid. This was followed by free draughts of warm water, until only a faint odor of sassafras was discoverable. The vomiting relieved him and he was soon restored to consciousness. He felt no discomfort except a sense of weakness and exhaustion, and was soon able to give the following account of himself: His employer having gone home, he was preparing to close up the office, when he espied a bottle of the oil of sassafras which had been left on the desk. Remembering that sassafras had been recommended for the removal of an eru tion that disfigured his face, he thought this a good opportunity for giving it a trial, and turning up the bottle—to use his own language—he took two large swallows of its contents. In a few minutes he began to feel very *stiff*, as he expressed it, but proceeded to close up the shutters preparatory to leaving for home. He raised the window for this purpose, but had not strength to hold it in this position, and it dropped from his grasp, and at the same time he fell to the floor unconscious. This suggestive case led me to make numerous experiments on the lower animals with very interesting results. Ten drops of the oil were injected hypodermically under the skin of a mouse. The animal quickly succumbed and died convulsed. By repeated experiments I was able so to regulate the dose as to get the characteristic effects of the drug without causing the speedy death of the mouse. A glass rod was dipped into the oil and held in front of the mouse, and he seized it with his mouth. This was repeated at intervals of a few minutes, until a sufficient quantity was taken to produce the desired effect. The first symptoms observed when a small quantity was thus taken, was a slight convulsive movement, which was repeated at intervals of a few seconds, and agitated the animal's body very much like a severe hiccough. This gradually increased in severity, the movements became more unsteady, the body more arched, and the limbs so stiff that the mouse stood on tiptoe. It was noted that the one idea of escaping from the trap still predominated over all else, as he continued to climb up on the bars of the cage, only to fall on his side or back at each convulsion, until no longer able to rise.

I have repeated these experiments many times with great uniformity of result. Sometimes they would dance about for half an hour, with a peculiar convulsive movement that would jerk the head and front feet from the table. Again they would fall on their side with each convulsion and regain their feet immediately only to repeat the same movement. With cats and dogs the result was somewhat different. A drachm under the skin of a cat caused such profound insensibility that she was supposed to be dead, and thrown away, but it seems that only one of the reputed nine lives of the animal had been reached, as the next day she turned up none the worse for the experiment. A full-grown dog was paralyzed in his hind legs by a similar dose hypodermically over the loins, but it recovered. Many other experiments might be adduced, but I will not trespass on your time. There is one other property possessed by this drug that is worthy of mention,—it is a germicide and anti-ferment of no mean quality. In some clumsy experiments made by myself I have estimated its potency in this field as about one-half the strength of carbolic acid. It has long been used as a domestic remedy for the destruction of lice and other vermin."

For some years past I have had an intention of bringing before the profession reasons, rather feeble it must be admitted, for the supposition that the medicine under consideration has marked potency in a direction, so far as I know, not suspected by medical men. Up to this time the declaration on the part of standard writers that sassafras is a remedy of questionable power, and the fact that it is hawked about the streets and used freely as a tea all over the country, have caused me to refrain from bringing before a scientific body my limited experience presently to be detailed. But the recent declaration that this drug possesses toxic properties may justify me in making the following statement.

Years ago I was called to a woman among the poorer classes, of good intelligence and education, who was having a miscarriage. Upon my inquiring as to the cause of the mishap, with a prefatory reference to her poverty and already large family, she stated that she had induced the abortion herself—that she had done so on previous occasions. She had employed, she said, "what other women used," sassafras tea. She was surprised that I did not know of the property of sassafras as an oxytoxic. She spoke as if all her friends knew how to use it as an ecbolic, and she evidently looked upon it as a specific. Tea, she said, made from four or five pieces of the root, as large as the thumb and twice as long, would produce abortive effect.

A year or two later I was called to a woman two months pregnant. For several days she had had symptoms of miscarriage of so pronounced a character that arrest of the process was doubtful. I found the patient very anxious to have a child; she disclaimed the intention of inducing abortion, and to all my inquiries as to a possible cause, of the hæmorrhage she gave answers which left me no further question except this: "Have you been drinking sassafras tea?" Surprised, she replied that for a week past she had used it at breakfast and supper. The proper remedies for her condition were prescribed, the possibly offending tea left off, and in twenty-four hours all was quiet in utero.

Farther than this my experience with sassafras as a possible abortifacient does not extend; possibly some one present can supplement my remarks with knowledge or experience of his own.

A study of the toxic effects of sassafras as reported by Dr. Hill, and here suggested, would seem to show a triple resemblance to three familiar articles, opium, strychnine and ergot.

In its action as a narcotic and sudorific it resembles opium.

In its property of inducing tetanic and clonic spasms, followed by paralysis, it is similar to strychnine.

In its power hinted at of exciting the uterus, it may be likened to ergot.

It may be of interest here to call attention to the fact that the first reference to the use of ergot as an ecbolic was made by Stearns in 1807, whereas it had been used by midwives certainly as early as 1688, and probably very much earlier.

482 North Clark Street.

